# Quality by design: using intelligent forms to ensure study protocol compliance and participant safety

**DOI:** 10.1186/1745-6215-16-S2-P31

**Published:** 2015-11-16

**Authors:** Rejive Dayanandan, Elizabeth Wincott, Michael Lay, Richard Haynes, Martin Landray, Jane Armitage

**Affiliations:** 1 University of Oxford, Oxford, UK

## Background

Ensuring compliance with the study protocol forms the cornerstone of any clinical trial.

## Methods

‘Intelligent’ electronic case report forms (eCRFs) can allow built-in checks to ensure the Study Protocol is adhered to. For a large, complex, international randomized trial a bespoke eCRF was deployed on laptops provided to study sites which enforced checks at screening of age, previous medical history, concomitant medication and safety blood (ALT, creatinine and CK within range on a desk-top analyser) to ensure that only those eligible entered a pre-randomization run-in and were issued drug. Following an active Run-in, the eCRF ensured that only those still eligible based on updated medication, blood safety checks and the absence of contraindications could be randomized. During the study blood safety checks were mandated and results outside pre-specified ranges prompted additional checks.

## Results

The eCRF ensured only eligible participants were randomized. In addition, during the study a higher than expected rate of statin-associated myopathy in Chinese participants was noted. The Protocol design mandated CK measurement when muscle pain or weakness was reported but, in light of the increased risk of myopathy, was amended and the eCRF adapted to ensure research coordinators measured CK if a participant's routinely-measured ALT was > 1.5xULN, irrespective of muscle symptoms. This approach to ensuring appropriate safety checks resulted in significant numbers of participants being identified as being at risk of myopathy before developing muscle symptoms.

## Conclusion

‘Intelligent’ eCRFs can ensure that the Protocol is followed and enhance the quality of study data and participant safety.

